# Supplementary material for: Polarization and Charge-Separation of Moiré Excitons in van der Waals Heterostructures
Source: Nano Lett. 2024 Nov 11;24(46):14702–8. doi: 10.1021/acs.nanolett.4c03915 (PMC11583365; doi:10.1021/acs.nanolett.4c03915)
Supplement: Supplementary file 1 — nl4c03915_si_001.pdf [file nl4c03915_si_001.pdf]

Supplemental information: Polarization and charge-separation of moiré excitons in van der Waals heterostructures

Joakim Hagel,[1] Samuel Brem,[2] and Ermin Malic[2,1,*]

[1]Department of Physics, Chalmers University of Technology, 412 96 Gothenburg, Sweden
[2]Department of Physics, Philipps University of Marburg, 35037 Marburg, Germany

I. ATOMIC RECONSTRUCTION

When the twist angle is small the lattice no longer remains rigid, but instead undergoes a process known as atomic reconstruction [1, 2]. Here, the lattice relaxes in order minimize the local stacking energies. Consequently, large domains form which are separated via thin domain walls, which in R-type stacking takes the form of triangular domains [2]. This deformation of the rigid lattice fundamentally changes the geometry and depth of the moiré potential [3]. It is therefore essential to model the relaxation of the lattice in order to gain microscopic insights into the reconstructed moiré potential. The atomic reconstruction can be modeled by setting up an integral for the total stacking energy within a continuum model [3, 4]

$$\mathcal{E} = \int_{\mathcal{A}_\mathrm{M}} d^2\mathbf{r}\Big[\sum_l \mathcal{U}^l + W_{R/H}(\mathbf{r}_0)\Big], \tag{1}$$

where l is the layer index, $\mathcal{U}^l$ the elastic energy and $W_{R/H}(\mathbf{r}_0)$ the adhesion energy between the layers. Here, $\mathbf{r}_0 = \theta\hat{z}\times\mathbf{r} + \mathbf{u}^t(\mathbf{r}) - \mathbf{u}^b(\mathbf{r})$, where $\mathbf{u}(\mathbf{r})$ is the displacement vector responsible for the atomic reconstruction. The elastic energy $\mathcal{U}^l$ is given by

$$\mathcal{U}^l = \frac{\lambda^l}{2}(u^l_{i,i})^2 + \mu^l u^l_{i,j}u^l_{j,i}, \tag{2}$$

where λ and μ are the material-specific Lamé parameters [5]. Here, we have assumed only small displacements, so that the displacement vector $\mathbf{u}(\mathbf{r})$ can be related to the linear strain tensor as $\varepsilon_{ij} = \frac{1}{2}(u_{i,j} + u_{j,i})$ with $u_{i,j} = \frac{1}{2}(\partial_i u_j + \partial_j u_i)$ and $i(j) = (x, y)$. Furthermore, the adhesion energy between the layers $W_{R/H}(\mathbf{r}_0)$ is given by

$$\begin{aligned} W_{R/H}(\mathbf{r}_0) &= -\kappa\mathcal{Z}^2_{R/H}(\mathbf{r}_0) \\ &+ \sum_{n=0}^{2}\Big[a_1\cos(\mathbf{G}_n\mathbf{r}_0) + a_2\sin(\mathbf{G}_n\mathbf{r}_0 + \gamma_{R/H})\Big], \end{aligned} \tag{3}$$

where $\mathbf{G}_n$ are Brillouin-zone lattice vectors and $\mathcal{Z}^2_{R/H}(\mathbf{r}_0)$ is the term corresponding to the interlayer distance relaxation and is given by

$$\begin{aligned} \mathcal{Z}_{R/H}(\mathbf{r}_0) &= \frac{1}{2\kappa}\sum_{n=0}^{2}\Big[a_1 A\cos(\mathbf{G}_n\mathbf{r}_0) \\ &+ a_2|\mathbf{G}_n|\sin(\mathbf{G}_n\mathbf{r}_0 + \gamma_{R/H})\Big]. \end{aligned} \tag{4}$$

Here, κ, a_1, a_2 and A are parameters fitted from density functional theory (DFT) and obtained from Ref.[4]. By expanding the displacement vectors as a Fourier series $\mathbf{u}^l(\mathbf{r}) = \sum_n \mathbf{u}^l_n e^{i\mathbf{g}_n\mathbf{r}}$, where $\mathbf{g}_n$ are the reciprocal moiré vectors, we go from an integral problem depending on $\mathbf{u}^l(\boldsymbol{r})$ to an optimization problem depending on the Fourier coefficients $\mathbf{u}^l_n$, which can then be numerically calculated [3].

II. MOIRÉ POTENTIAL

In the twisted $MoSe_2$-WSe_2 heterostructure, we have three components to the moiré potential for the low-lying KK interlayer excitons [6]. Since the tunneling is very weak around the K-point in R-type stacked $MoSe_2$-WSe_2 heterostructures [7], we can neglect this component to the moiré potential. In a atomically reconstructed lattice, the dominating component is instead the scalar strain [1], directly obtained from the displacement vectors

$$S^l_\lambda(\mathbf{r}) = \sum_i u^l_{i,i}(\mathbf{r})g^l_\lambda, \tag{5}$$

where $\lambda = (c, v)$ is the compound band index and g^l_λ is the gauge factor determining band edge variation as obtained from DFT [8].

In addition to the scalar strain, we also have a smaller component stemming from atomic rotation called the piezo potential [4]. The polarization induced by the piezo charges can be directly obtained from the displacement vectors [3, 4]

$$\mathcal{P}^l(\mathbf{r}) = e^l_{11}(u^l_{x,x} - u^l_{y,y}, -2u^l_{x,y}). \tag{6}$$

Here, e^l_{11} is the material-specific piezo coefficient [9]. By using the Gauss law and solving the Poisson equation we can calculate the resulting band edge variation from the piezo potential [3].

The last component of the moiré potential is the stacking-dependent polarization induced shift (alignment shift) of the band edges. By extracting the shift for each

* ermin.malic@chalmers.se

high-symmetry stacking from DFT and smoothly interpolating between them [6, 10] we can map out the total potential. Furthermore, by adding the displacement vectors we can deform the geometry of the potential as expected from the the atomic reconstruction [3]. The final formula for the alignment shift is then given by

$$A_l^\lambda(\mathbf{r}) = \mathrm{Re}\Big[v_l^\lambda + (\mathcal{A}_l^\lambda + \mathcal{B}_l^\lambda e^{i2\pi/3}) \sum_{n=0}^{2} e^{i(\mathbf{g}_n\cdot\mathbf{r}+\mathbf{G}_n\cdot\Delta\mathbf{u}^l(\mathbf{r}))}\Big]. \tag{7}$$

where v_l^λ, $\mathcal{A}_l^\lambda$ and $\mathcal{B}_l^\lambda$ are parameters which is obtained as DFT input [6]. Here, $\Delta\mathbf{u}(\mathbf{r}) = \mathbf{u}^t(\mathbf{r}) - \mathbf{u}^b(\mathbf{r})$ are the displacement vector of the atomic lattice, giving rise to the deformation of the potential.

Since all moiré potential components act as a renormalization on the band structure we can merge them into one total moiré potential, and then expend it as a Fourier series

$$V_l^\lambda(\mathbf{r}) = S_\lambda^l(\mathbf{r}) + P^l(\mathbf{r}) + A_l^\lambda(\mathbf{r}) = \sum_{\mathbf{g}} m_{\boldsymbol{g}}^{l\lambda} e^{i\mathbf{g}\cdot\mathbf{r}}, \tag{8}$$

where $S_\lambda^l(\mathbf{r})$ is the scalar strain potential, $P^l(\mathbf{r})$ the piezo potential, $A_l^\lambda(\mathbf{r})$ the alignment shift, and $V_l^\lambda(\mathbf{r})$ is the total moiré potential. Furthermore, $m_{\boldsymbol{g}}^{l\lambda}$ are the Fourier coefficients of the total moiré potential as obtained from

$$m_{\boldsymbol{g}}^{l\lambda} = \frac{1}{\mathcal{A}_\mathrm{M}} \int_{\mathcal{A}_\mathrm{M}} d\mathbf{r} e^{-i\mathbf{g}\cdot\mathbf{r}} V_l^\lambda(\mathbf{r}) \tag{9}$$

with $\mathcal{A}_\mathrm{M}$ as the moiré unit cell area.

III. GENERALIZED EIGENVALUE EQUATION

In order to model the exciton energy landscape with charge separation in an atomically reconstructed lattice we first set up a Hamiltonian in second quantization in momentum space

$$H = \sum_{\boldsymbol{k}\lambda} \varepsilon_{\boldsymbol{k}}^\lambda \lambda_{\boldsymbol{k}}^\dagger \lambda_{\boldsymbol{k}} + \sum_{\boldsymbol{kq}\lambda} M_{\boldsymbol{q}}^\lambda \lambda_{\boldsymbol{k+q}}^\dagger \lambda_{\boldsymbol{k}} + \sum_{\boldsymbol{kk'q}} V_{\boldsymbol{q}}^{cv} c_{\boldsymbol{k+q}}^\dagger v_{\boldsymbol{k'-q}}^\dagger v_{\boldsymbol{k'}} c_{\boldsymbol{k}}, \tag{10}$$

where the momentum of the electron/hole is given by $\boldsymbol{k}^{(\prime)}$, while $\boldsymbol{q}$ denotes the transferred momentum. The electron (hole) dispersion is denoted by $\varepsilon_{\boldsymbol{k}}^{c(v)}$ and the Fourier-transformed moiré potential by $M_{\boldsymbol{q}}^\lambda$. Furthermore, the Coulomb matrix element responsible for the formation of excitons is given by $V_{\boldsymbol{q}}^{cv}$, where the generalized Keldysh potential has been used [11]. Moreover, $c^{(\dagger)}$ and $v^{(\dagger)}$ are the annihilation (creation) operators for the conduction and the valance band, respectively. Since electrons now remain fixed in the molybdenum layer and holes in the tungsten layer, we have dropped the layer index from the equation.

The general two-particle exciton state reads $|X\rangle = \sum_{\boldsymbol{kk'}} \Psi_{\boldsymbol{kk'}} c_{\boldsymbol{k}}^\dagger v_{\boldsymbol{k'}} |0\rangle = X^\dagger |0\rangle$, where $\Psi_{\boldsymbol{kk'}}$ is the general two-particle wave function and $X^{(\dagger)}$ the exciton annihilation (creation) operator. By acting the Hamiltonian upon the general exciton state we can derive an eigenvalue problem $H|X\rangle = E|X\rangle$, which then reads

$$(\varepsilon_{\boldsymbol{k}}^c - \varepsilon_{\boldsymbol{k'}}^v)\Psi_{\boldsymbol{kk'}} + \sum_{\boldsymbol{q}} \Big(M_{\boldsymbol{q}}^c \Psi_{\boldsymbol{k-q},\boldsymbol{k'}} - M_{\boldsymbol{q}}^v \Psi_{\boldsymbol{k},\boldsymbol{k'+q}}\Big) - \sum_{\boldsymbol{q}} V_{\boldsymbol{q}}^{cv} \Psi_{\boldsymbol{k-q},\boldsymbol{k'-q}} = E\Psi_{\boldsymbol{kk'}}, \tag{11}$$

where E is the exciton energy.

By introducing the the center-of-mass momentum (COM) $\boldsymbol{Q} = \boldsymbol{k}_c - \boldsymbol{k}_v = \boldsymbol{k}_e + \boldsymbol{k}_h$ we can map the dependence on the momentum from $\boldsymbol{k}/\boldsymbol{k'}$ to $\boldsymbol{k}/Q$. Here, we have introduced the notation $\tilde{\Psi}_{\boldsymbol{kQ}} = \Psi_{\boldsymbol{k},\boldsymbol{k-Q}} \leftrightarrow \Psi_{\boldsymbol{kk'}} = \tilde{\Psi}_{\boldsymbol{k},\boldsymbol{k-k'}}$, which translates between the pictures. Now applying the zone-folding technique we restrict the summation over $\boldsymbol{k}/\boldsymbol{Q}$ to the first mBZ (mini-Brillouin zone) and mBZ lattice vectors $\boldsymbol{g}(\tilde{\boldsymbol{g}})$ as $\tilde{\Psi}_{\boldsymbol{k+g},\boldsymbol{Q}+\tilde{\boldsymbol{g}}} = \Phi_{\boldsymbol{kQ}}(\boldsymbol{g},\tilde{\boldsymbol{g}})$. Note that the moiré potential is periodic such that $M_{\boldsymbol{q}}^\lambda = \sum_{\boldsymbol{g}} m_{\boldsymbol{g}}^\lambda \delta_{\boldsymbol{q},\boldsymbol{g}}$, where $m_{\boldsymbol{g}}^\lambda$ are the Fourier coefficients obtained from Eq. 9. Consequently, we can derive the zone-folded eigenvalue problem as

$$\Delta\varepsilon_{\boldsymbol{kQ}}^{cv}(\boldsymbol{g},\tilde{\boldsymbol{g}})\Phi_{\boldsymbol{k}}^{\boldsymbol{Q}}(\boldsymbol{g},\tilde{\boldsymbol{g}}) + \sum_{\boldsymbol{g'}\tilde{\boldsymbol{g}}'} \Big(m_{\tilde{\boldsymbol{g}}-\tilde{\boldsymbol{g}}'}^c \delta_{\boldsymbol{g}-\boldsymbol{g'},\tilde{\boldsymbol{g}}-\tilde{\boldsymbol{g}}'} - m_{\tilde{\boldsymbol{g}}-\tilde{\boldsymbol{g}}'}^v \delta_{\boldsymbol{g}-\boldsymbol{g'},\tilde{\boldsymbol{g}}-\tilde{\boldsymbol{g}}'}\Big)\Phi_{\boldsymbol{k}}^{\boldsymbol{Q}}(\boldsymbol{g'},\tilde{\boldsymbol{g}}') - \sum_{\boldsymbol{pg'}} V_{\boldsymbol{k-p+g-g'}}^{cv} \Phi_{\boldsymbol{k}}^{\boldsymbol{Q}}(\boldsymbol{g'},\tilde{\boldsymbol{g}}) = E_{\boldsymbol{Q}}\Phi_{\boldsymbol{k}}^{\boldsymbol{Q}}(\boldsymbol{g},\tilde{\boldsymbol{g}}), \tag{12}$$

where $\Phi_{\boldsymbol{k}}^{\boldsymbol{Q}}(\boldsymbol{g},\tilde{\boldsymbol{g}})$ is the zone-folded two-particle wave function and $E_{\boldsymbol{Q}}$ are the exciton energies as a function of COM momentum. Here, $\Delta\varepsilon_{\boldsymbol{kQ}}^{cv}(\boldsymbol{g},\tilde{\boldsymbol{g}}) = \varepsilon_{\boldsymbol{k+g}}^c - \varepsilon_{\boldsymbol{k+g-Q}-\tilde{\boldsymbol{g}}}^v$ is the difference between the zone-folded conduction/valance band dispersion. By treating the eigenvalue problem as a sparse matrix to be diagonalized we can calculate the exciton energies. This was done using the c++ library Armadillo [12, 13], where the number of moiré shells for $\boldsymbol{g}$ was converged at 7 and for $\tilde{\boldsymbol{g}}$ it was chosen as $N_{shell}^{el} \approx \frac{\boldsymbol{k}_{cut}^{el}}{|\boldsymbol{g}_1|}$ such that the number of shells for the electron momentum zone-folding was dynamic with the change in twist angle. Here, we have introduced $\boldsymbol{k}_{cut}^{el} = \boldsymbol{k}_{max} + \boldsymbol{g}_{max}$, where the energies $E_{\boldsymbol{Q}}$ were converged at $\boldsymbol{k}_{max} \approx 2.2\mathrm{nm}^{-1}$.

IV. CHARGE DENSITIES

After diagonalizing the generalized moiré exciton eigenvalue problem in Eq. 12 we want to gain access to to the spatial distribution of electrons and holes. This is done by expanding electron densities in real space

$\rho^e(\boldsymbol{r}) = \langle X_{\boldsymbol{Q}}|\Psi_e^\dagger(\boldsymbol{r})\Psi_e(\boldsymbol{r})|X_{\boldsymbol{Q}}\rangle$ with the exciton state

$$|X_{\boldsymbol{Q}}\rangle = \sum_{\boldsymbol{k}\boldsymbol{g}\tilde{\boldsymbol{g}}} \Phi_{\boldsymbol{k}}^{\boldsymbol{Q}}(\boldsymbol{g},\tilde{\boldsymbol{g}}) c_{\boldsymbol{k}+\boldsymbol{g}}^\dagger v_{\boldsymbol{k}+\boldsymbol{g}-\boldsymbol{Q}-\tilde{\boldsymbol{g}}} |0\rangle\,, \quad (13)$$

where $\Psi_\lambda^\dagger(\boldsymbol{r}) = \sum_{\boldsymbol{p}} e^{i\boldsymbol{p}\cdot\boldsymbol{r}}\lambda^\dagger$. Simplifying the the expression for electron densities we obtain

$$\rho^e(\boldsymbol{r}) = \sum_{\Delta} e^{i\Delta\cdot\boldsymbol{r}} \sum_{\boldsymbol{k}\boldsymbol{g}\tilde{\boldsymbol{g}}} \Phi_{\boldsymbol{k}}^{\boldsymbol{Q}*}(\boldsymbol{g}+\Delta,\tilde{\boldsymbol{g}}+\Delta)\Phi_{\boldsymbol{k}}^{\boldsymbol{Q}}(\boldsymbol{g},\tilde{\boldsymbol{g}}) \quad (14)$$

with $\Delta = \boldsymbol{g}-\boldsymbol{g}'$. In analogy, we can also calculate the hole density $\rho^h(\boldsymbol{r}) = \langle 0|\Psi_v^\dagger(\boldsymbol{r})\Psi_v(\boldsymbol{r})|0\rangle$ $- \langle 0|X_{\boldsymbol{Q}}\Psi_v^\dagger(\boldsymbol{r})\Psi_v(\boldsymbol{r})X_{\boldsymbol{Q}}^\dagger|0\rangle$ yielding

$$\rho^h(\boldsymbol{r}) = \sum_{\Delta} e^{i\Delta\cdot\boldsymbol{r}} \sum_{\boldsymbol{k}\boldsymbol{g}\tilde{\boldsymbol{g}}} \Phi_{\boldsymbol{k}}^{\boldsymbol{Q}*}(\boldsymbol{g},\tilde{\boldsymbol{g}}+\Delta)\Phi_{\boldsymbol{k}}^{\boldsymbol{Q}}(\boldsymbol{g},\tilde{\boldsymbol{g}}). \quad (15)$$

By introducing the relative $\boldsymbol{r} = \boldsymbol{r}_e - \boldsymbol{r}_h$ and center-of-mass coordinates $\boldsymbol{R} = \frac{1}{\mu}(m_e\boldsymbol{r}_e + m_h\boldsymbol{r}_h)$, where μ is the reduced mass, we can set up an expression for the conditional electron-hole densities

$$\begin{aligned} P_{eh}(\boldsymbol{r},\boldsymbol{R}) &= \langle \boldsymbol{Q}|\Psi_e^\dagger(\boldsymbol{R}+\beta\boldsymbol{r})\Psi_e(\boldsymbol{R}+\beta\boldsymbol{r}) \\ &\quad \times\Psi_h^\dagger(\boldsymbol{R}-\alpha\boldsymbol{r})\Psi_h(\boldsymbol{R}-\alpha\boldsymbol{r})|\boldsymbol{Q}\rangle \\ &= \langle 0|X_{\boldsymbol{Q}}\Psi_e^\dagger(\boldsymbol{R}+\beta\boldsymbol{r})\Psi_e(\boldsymbol{R}+\beta\boldsymbol{r}) \\ &\quad \times\Psi_h^\dagger(\boldsymbol{R}-\alpha\boldsymbol{r})\Psi_h(\boldsymbol{R}-\alpha\boldsymbol{r})X_{\boldsymbol{Q}}^\dagger|0\rangle, \end{aligned} \quad (16)$$

where $\alpha(\beta) = m_{e(h)}/(m_e+m_h)$ and $P_{eh}(\boldsymbol{r},\boldsymbol{R}) = |\bar{\Psi}_{\boldsymbol{Q}}(\boldsymbol{r},\boldsymbol{R})|^2$ corresponds to the probability. Following the same approach, we can derive an expression for $P_{eh}(\boldsymbol{r},\boldsymbol{R})$ reading

$$P_{eh}(\boldsymbol{r},\boldsymbol{R}) = \sum_{\substack{\boldsymbol{k}\boldsymbol{g}\tilde{\boldsymbol{g}} \\ \boldsymbol{k}'\boldsymbol{g}'\tilde{\boldsymbol{g}}'}} e^{i(\tilde{\boldsymbol{g}}-\tilde{\boldsymbol{g}}')\cdot\boldsymbol{R})} e^{-i(\tilde{\boldsymbol{g}}-\tilde{\boldsymbol{g}}')\cdot\alpha\boldsymbol{r}} \times \Phi_{\boldsymbol{k}}^{\boldsymbol{Q}*}(\boldsymbol{g},\tilde{\boldsymbol{g}})\Phi_{\boldsymbol{k}}^{\boldsymbol{Q}*}(\boldsymbol{g}',\tilde{\boldsymbol{g}}'). \quad (17)$$

By now introducing the unfolded two-particle wave function $\tilde{\Phi}_{\boldsymbol{Q}}(\boldsymbol{k}+\boldsymbol{g},\tilde{\boldsymbol{g}}) = \Phi_{\boldsymbol{k}}^{\boldsymbol{Q}}(\boldsymbol{g},\tilde{\boldsymbol{g}})$ and taking the trace of the relative electron-hole coordinates, i.e integrating over $\boldsymbol{r}$ $P_{eh}(\boldsymbol{R}) = \frac{1}{A}\int d^2r P_{eh}(\boldsymbol{r},\boldsymbol{R})$, the following expression for the COM density is obtained

$$P_{eh}(\boldsymbol{R}) = \sum_{\Delta'} e^{i\Delta'\cdot\boldsymbol{R}} \sum_{\boldsymbol{k}\tilde{\boldsymbol{g}}} \tilde{\Phi}_{\boldsymbol{Q}}^*(\boldsymbol{k}+\alpha\Delta,\tilde{\boldsymbol{g}}+\Delta)\tilde{\Phi}_{\boldsymbol{Q}}(\boldsymbol{k},\tilde{\boldsymbol{g}}) \quad (18)$$

with $\Delta' = \tilde{\boldsymbol{g}} - \tilde{\boldsymbol{g}}'$. By comparing Eq. 14, Eq. 15 and Eq. 18 we can now see that all densities can be rewritten as a function of the same form factor

$$\begin{aligned} \rho_e(\boldsymbol{r}) &= \sum_{\Delta} e^{i\Delta\cdot\boldsymbol{r}}\Gamma_{\boldsymbol{Q}}(\Delta,\Delta) \\ \rho_h(\boldsymbol{r}) &= \sum_{\Delta} e^{i\Delta\cdot\boldsymbol{r}}\Gamma_{\boldsymbol{Q}}(0,\Delta) \\ \rho_X(\boldsymbol{R}) &= \sum_{\Delta'} e^{i\Delta'\cdot\boldsymbol{R}}\Gamma_{\boldsymbol{Q}}(\alpha\Delta',\Delta'), \end{aligned} \quad (19)$$

where $\rho_X(\boldsymbol{R}) = P_{eh}(\boldsymbol{R})$. The form factor is given by

$$\Gamma_{\boldsymbol{Q}}(\boldsymbol{q},\Delta) = \sum_{\boldsymbol{k}\boldsymbol{G}} \tilde{\Phi}_{\boldsymbol{Q}}^*(\boldsymbol{k}+\boldsymbol{q},\boldsymbol{G}+\Delta)\tilde{\Phi}_{\boldsymbol{Q}}(\boldsymbol{k},\boldsymbol{G}). \quad (20)$$

[1] M. Van Winkle, I. M. Craig, S. Carr, M. Dandu, K. C. Bustillo, J. Ciston, C. Ophus, T. Taniguchi, K. Watanabe, A. Raja, *et al.*, Rotational and dilational reconstruction in transition metal dichalcogenide moiré bilayers, Nature Communications **14**, 2989 (2023).

[2] A. Weston, Y. Zou, V. Enaldiev, A. Summerfield, N. Clark, V. Zólyomi, A. Graham, C. Yelgel, S. Magorrian, M. Zhou, *et al.*, Atomic reconstruction in twisted bilayers of transition metal dichalcogenides, Nature Nanotechnology **15**, 592 (2020).

[3] J. Hagel, S. Brem, J. A. Pineiro, and E. Malic, Impact of atomic reconstruction on optical spectra of twisted tmd homobilayers, Phys. Rev. Mater. **8**, 034001 (2024).

[4] V. Enaldiev, V. Zólyomi, C. Yelgel, S. Magorrian, and V. Fal'ko, Stacking domains and dislocation networks in marginally twisted bilayers of transition metal dichalcogenides, Physical Review Letters **124**, 206101 (2020).

[5] F. Ferreira, S. Magorrian, V. Enaldiev, D. Ruiz-Tijerina, and V. Fal'ko, Band energy landscapes in twisted homobilayers of transition metal dichalcogenides, Applied Physics Letters **118**, 241602 (2021).

[6] S. Brem, C. Linderälv, P. Erhart, and E. Malic, Tunable phases of moiré excitons in van der waals heterostructures, Nano Letters **20**, 8534 (2020), pMID: 32970445, https://doi.org/10.1021/acs.nanolett.0c03019.

[7] J. Hagel, S. Brem, C. Linderälv, P. Erhart, and E. Malic, Exciton landscape in van der waals heterostructures, Phys. Rev. Research **3**, 043217 (2021).

[8] Z. Khatibi, M. Feierabend, M. Selig, S. Brem, C. Linderälv, P. Erhart, and E. Malic, Impact of strain on the excitonic linewidth in transition metal dichalcogenides, 2D Materials **6**, 015015 (2018).

[9] H. Rostami, F. Guinea, M. Polini, and R. Roldán, Piezoelectricity and valley chern number in inhomogeneous hexagonal 2d crystals, npj 2D Materials and Applications **2**, 15 (2018).

[10] J. Hagel, S. Brem, and E. Malic, Electrical tuning of moiré excitons in $MoSe_2$ bilayers, 2D Materials **10**, 014013 (2023).

[11] D. Erkensten, S. Brem, R. Perea-Causín, and E. Malic, Microscopic origin of anomalous interlayer exciton transport in van der waals heterostructures, Physical Review Materials **6**, 094006 (2022).

[12] C. Sanderson and R. Curtin, Practical sparse matrices in c++ with hybrid storage and template-based expression optimisation, Mathematical and Computational Applications **24**, 70 (2019).

[13] C. Sanderson and R. Curtin, Armadillo: a template-based c++ library for linear algebra, Journal of Open Source Software **1**, 26 (2016).
